# The *Leishmania* Skin Test Predicts Clinic-Immunologic and Therapeutic Outcomes in Cutaneous Leishmaniasis

**DOI:** 10.3390/pathogens13111018

**Published:** 2024-11-19

**Authors:** Luiz H. Guimarães, Evelyn Zacarias, Sandra T. Nolasco, Almério N. Filho, Jamile Lago, Paulo R. L. Machado, Joyce Oliveira, Lucas P. Carvalho, Augusto Carvalho, Edgar M. Carvalho, Sérgio Arruda

**Affiliations:** 1 Immunology Service, University Hospital Professor Edgar Santos, Federal University of Bahia, Salvador 40110-160, Bahia, Brazil; luizhenriquesg@yahoo.com.br (L.H.G.); sandranolasco.derma@gmail.com (S.T.N.); jamilelago27@gmail.com (J.L.); 9pmachado@gmail.com (P.R.L.M.); carvalholp76@gmail.com (L.P.C.); augustomarcelino1@hotmail.com (A.C.); 2 Health Sciences Center, Santo Antonio de Jesus campus, Federal University of Recôncavo da Bahia, Santo Antônio de Jesus 44574-490, Bahia, Brazil; 3 National Institute of Science and Technology of Tropical Diseases (INCT-DT), MCTI CNPq, Salvador 40110-160, Bahia, Brazil; 4 Instituto Gonçalo Moniz, Fundação Oswaldo Cruz, Salvador 40296-710, Bahia, Brazil; evelyn.zacarias@fiocruz.br (E.Z.); almerio2001@gmail.com (A.N.F.); joyce.oliveira@fiocruz.br (J.O.); 5 Faculty of Pharmacy, Ondina Campus, Federal University of Bahia, Salvador 40170-115, Bahia, Brazil; 6 Department of Life Sciences, School of Medicine, Salvador Campus, State University of Bahia, Salvador 41200-105, Bahia, Brazil

**Keywords:** cutaneous leishmaniasis, *L. braziliensis*, delayed-type hypersensitivity negative, pathology, Granzyme

## Abstract

Cutaneous leishmaniasis (CL), caused by *Leishmania braziliensis*, is closely associated with a severe form of the disease, indicated by a positive *Leishmania* skin test (LST) that assesses and reflects the presence of immune T cells specific to *Leishmania* antigens. In this study, we compare the clinical, immunologic, and histopathologic features between *Leishmania* skin test-positive (LST+) and *Leishmania* skin test-negative (LST-) in CL. Compared to LST+ patients, LST- patients had larger lesions and had been sicker for longer, presented with more instances of therapeutic failure with meglumine antimonate, (MA) and the healing times were higher than LST+. While granulomas were less frequent and the parasite load was higher in LST-, there were more CD8+ T cells and an enhanced production of Granzyme B in the supernatants of biopsies from LST- subjects. This study shows that in LST-, an impairment in Th1 immune response is associated with a high parasite burden, and the pathology is mediated by CD8+ T cells and the enhanced production of Granzyme B. The abnormalities in the immunologic response in LST- patients lead to a more severe disease with a high rate of failure to therapy.

## 1. Introduction

Cutaneous leishmaniasis (CL) is a disease caused by intracellular parasites of the *Leishmania* genus. *Leishmania (Viannia) braziliensis* is the most prevalent causal agent of the disease in the Americas [1,2,3]. *Leishmania* species are transmitted by the bite of infected female sandflies, which transform into amastigotes and replicate inside macrophages. Macrophages are activated by interferon-γ (IFN-γ) [4,5,6], produced by a T helper 1 cell (Th1). This immune response is important to decrease parasite multiplication [7,8]. However, despite this protective immune response, the parasite survives, and a chronic and potent inflammatory reaction leads to ulcer development [7,8,9,10].

In 1926, Montenegro described the intradermal test for diagnosing leishmaniasis ulcers [11]. This skin test induces a delayed type of hypersensitivity reaction to *Leishmania* antigens, and a positive LST shows a high sensitivity for the diagnosis of American Tegumentary Leishmaniasis (ATL) [12]. It is known that the LST assesses and reflects the presence of immune T cells specific to *Leishmania* antigens. This immune response recruits inflammatory cells to the dermis, which leads to induration that can be measured. Induration larger than 5 mm is considered positive for a *Leishmania* infection. Moreover, the LST has also been used to identify individuals who, despite being infected by *Leishmania* parasites, do not develop the disease and are considered as having an asymptomatic or subclinical infection [13,14].

The pathogenesis of leishmaniasis is dependent on parasite and host immunologic factors. In subjects infected with *L. infantum* who have an impaired Th1 immune response, parasites proliferate and disseminate, causing visceral leishmaniasis [15]. In patients mainly infected with *L. amazonensis* in the New World, a poor T cell response is also observed, and they develop diffuse CL [16]. In *L. braziliensis* infection, isolates of the same species may cause different clinical forms of the disease, such as CL, mucosal leishmaniasis, and disseminated leishmaniasis (DL) [17]. However, there is no evidence that these different clinical forms are associated with impairments in the Th1 immune responses [7,18]. A strong Th1 immune response is characteristic of an *L. braziliensis* infection. Even in patients who present DL with more than 1000 lesions, the inflammatory response is upregulated, and the pathogenesis of the diseases is linked to a decreased ability of macrophages in killing *Leishmania* [18,19]. In contrast with the role of CD4+ Th1 cells in controlling *Leishmania* infection, CD8+ T cells and NK cells as well as a high expression of IL-1β, IL-17, and Granzyme B (GzmB) at the lesion site are associated with pathology and ulcer development [9,20,21]. It is known that the activation of CD8+ T cells is dependent on CD4+T cells, but a small percentage of CL patients have an impairment in the Th1 immune response and have a negative LST [22]. We have previously shown that CL patients with LST- show a poor production of IFN-γ and TNF, and have a higher rate of therapeutic failure as compared to LST+ [23,24,25]. However, it is unclear how patients who have LST- develop ulcers that are caused by an enhanced inflammatory reaction. In the present study, using a large cohort, we evaluate the clinical, histopathologic, and immunologic features in CL patients with negative *Leishmania* skin test.

Furthermore, we evaluate and compare the tissue inflammatory response, cytokine profiles, and therapeutic outcomes in 134 patients with cutaneous leishmaniasis, stratified by their *Leishmania* skin test (LST) status, with 67 patients testing LST-positive (LST+) and 67 testing LST-negative (LST-). Patients with cutaneous leishmaniasis who are LST-positive will exhibit a distinct tissue inflammatory response and cytokine profile compared to LST-negative patients, which may be associated with differences in their therapeutic response. Specifically, LST-positive patients may demonstrate a more robust immune response and better therapeutic outcomes than LST- negative patients.

## 2. Materials and Methods

### 2.1. Study Design, Case Definition and Treatment

Participants in this prospective case–control study were 134 patients who presented suspicious ulcers of CL, half with LST- and half with LST+, whose CL diagnosis was confirmed by the presence of *L. braziliensis* DNA and the presence of amastigotes in lesion biopsies. The timeline for patient selection spanned from 2014 to 2021. All patients included in the study were HIV-negative, did not have chronic disability disease, and were not using immunosuppressive drugs. All patients attended and were treated at the Corte de Pedra Health Post (Supplementary Data S1).

Patients with LST+ and with LST- were treated with meglumine antimoniate (MA) and Glucantime (Sanofi Aventis, Paris, France,) with a dose of 20 mg/kg/day for 20 days. Patients were evaluated every 30 days for their response to the therapy. The cure rate was assessed 90 days after the initiation of therapy. The cure was defined as a complete reepithelization of the lesions, without raised borders. If a cure was not achieved, patients received a second course of MA for 30 days. If a cure was still not achieved, patients received a third course of MA for 30 days. If the cure was still not achieved after the third course of MA, patients were treated with Miltefosine 2.5 mg/kg/20 days. If a cure was not achieved after this, the patients were treated with amphotericin B with a dose of 3–5 mg/kg/day for 7 days (Supplementary Data S2) [26,27,28].

### 2.2. Leishmania Antigen and Intradermal Leishmania Skin Test

Soluble *Leishmania* antigen (SLA) was prepared as previously described and diluted in Phosphate-Buffered Saline (PBS) [12]. For the LST, 25 μg SLA in 0.1 mL was injected into the forearm, and induration was determined 48 h post-inoculation. A positive LST was considered when the induration was equal to or greater than 5 mm.

### 2.3. Histopathological Analysis

Biopsies of skin lesions were obtained using a 4 mm punch. Each biopsy was divided into three portions: one was placed in formalin for histopathological analysis, another was stored in RPMI 1640 medium (GIBCO BRL) to characterize the immune response at the lesion site, and the remaining portion was placed in RNA solution for PCR extraction. Biopsies were taken from the ulcer edges for PCR, histopathological, and immunohistochemical analysis, as well as to characterize the immune response at the lesion site. The control group consisted of randomly selected patients with a positive *Leishmania* skin test (LST+), matched by gender and age.

For the identification of granuloma, the percentage of inflammation and the necrosis area in the inflammation of the dermis of 26 LST+ and 26 LST- biopsies were randomly selected and matched by age and gender. The histopathologic analysis included the identification and quantification of the cells in the inflammatory infiltrate in the dermis. The quantification of inflammation and necrosis was performed by measuring images captured by a Moticam 1080 camera coupled to a Nikon Eclipse Ci microscope. The calculations were made in ImageJ (Bethesda, MD, USA). Amastigote counting was performed under the microscope in the analysis of immunostained biopsies.

### 2.4. Immunohistochemistry (IHC) and Double Staining

Electrostatically charged slides containing 4-micrometer tissue sections were subjected to immunohistochemistry for the detection of amastigotes, using a BALB/C polyclonal anti-*L*. *braziliensis* antibody kindly provided by Dr Hiro Goto from the University of São Paulo (made in-house); this had a titer of 1:2000, was revealed with MACH 1 Universal HRP-Polymer Detection (Biocare Medical, Pacheco, CA, USA, M1U539), and the double or simple immunostaining technique was applied using specific antibodies to identify and quantify the following markers: CD8+ (Abcam ab4055; 1:400), Granzyme B+ (Cell marque, Rocklin, CA, USA Ref.: 262A-16; 1:100), and amastigotes. CD8+ and GzmB+ cells were revealed by the DoubleStain IHC kit (Abcam, Cambridge, UK, ab210061). Simple immunostaining for *L. braziliensis* amastigotes was applied in 19 LST+ and 19 LST- biopsies for amastigotes counting under the microscope. Double immunostaining for CD8+ and GzmB+ cells was applied in the biopsies of 9 LST+ and 9 LST- lesions.

### 2.5. Cell Culture and Determination of Cytokines

The biopsies of *L. braziliensis* lesions from 11 LST+ and 11 LST- CL patients were randomly selected matched by gender and age and used for the characterization of the immune response on the ski; these were adjusted by weighing and were cultured in complete RPMI media without stimulus at 37 °C and 5% CO_2_ for 72 h. Supernatants were collected and stored at −20 °C until the time of cytokine quantification by the ELISA (R&D Systems, Minneapolis, MN, USA) sandwich method, according to the manufacturer’s instructions. The results are expressed as pg/mL.

### 2.6. Statistical Analyses

The clinical feature, histopathologic, and immunohistochemistry analysis were assessed via unpaired T-test, and the cure rate was calculated by Fisher’s exact test. The production of cytokines in the supernatants of the skin biopsies was evaluated by the Mann–Whitney Test. Survival analysis was performed by the Kaplan–Meier curve with Wilcoxon and log-rank tests. The results were considered significant when *p* < 0.05. Statistical analyses were performed using GraphPad (Boston, EUA) Prism 8 software.

## 3. Results

The clinical features and response to therapy in 134 patients, 67 with LST+ and an equal number with LST-, are shown in Table 1.

### 3.1. Clinical-Demographic Characteristics

**Figure 1:**
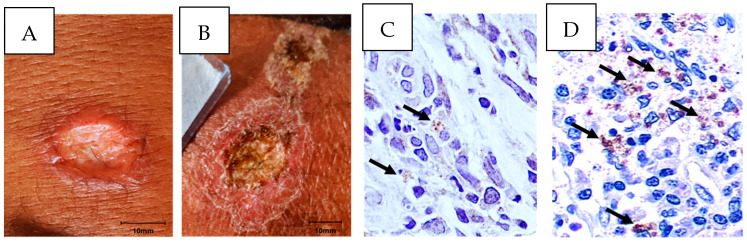
Clinical features of CL lesions and immunohistochemistry amastigotes detection. Clinical aspects of skin ulcers in LST+ (**A**) and LST- (**B**) patients. Detection of *L. braziliensis* amastigotes by immunohistochemistry in LST+ (**C**) and LST- (**D**) biopsies, in 100× magnification.

There was no difference among the groups regarding age and gender, but there were important differences related to the clinical features of the disease and response to therapy (Table 1). The illness duration was higher in LST- than in LST+ and the ulcers in these patients were large and with larger borders. The failure rate with MA was higher (56.7 vs. 34.3, *p* = 0.004) and the healing time was 1.6 times higher in LST- than LST+. The aspects of the ulcers and number of amastigotes in cells from LST- and LST+ are highlighted in Figure 1. The characteristics of the ulcers in the two groups of patients are shown in Figure 1A,B. An immunostaining for amastigotes displayed a lower number of amastigotes in LST+ than in LST- (Figure 1C,D).

**Figure 2:**
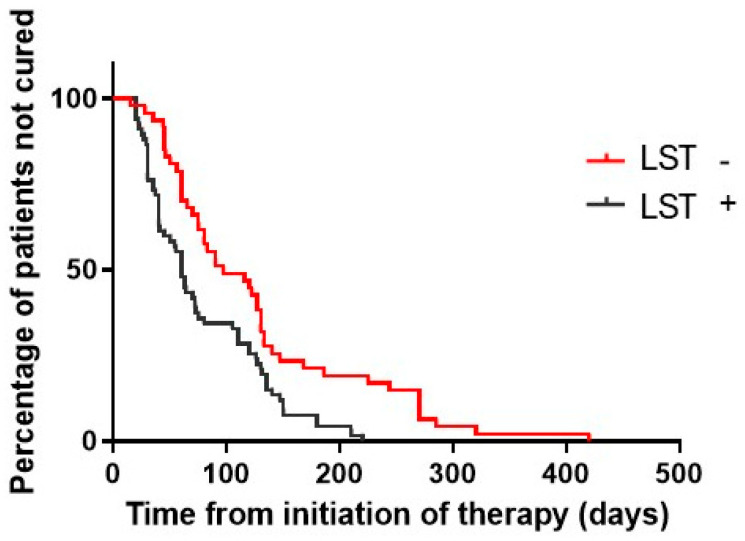
Kaplan–Meier estimates of the proportion of patients not cured in LST-positive and LST-negative subjects with cutaneous leishmaniasis. LST-positive (n = 67) and LST-negative (n = 47) patients were treated with meglumine antimoniate 20 mg/kg/day for 20 days. *p* = 0.0008 by log-rank test and *p* = 0.0016 by Wilcoxon test.

A Kaplan–Meier survival analysis was used to compare the differences in healing time between the two studied groups (*p* = 0.0008 by log-rank and *p* = 0.0016 by Wilcoxon test) (Figure 2).

### 3.2. Histopathological Features

**Figure 3:**
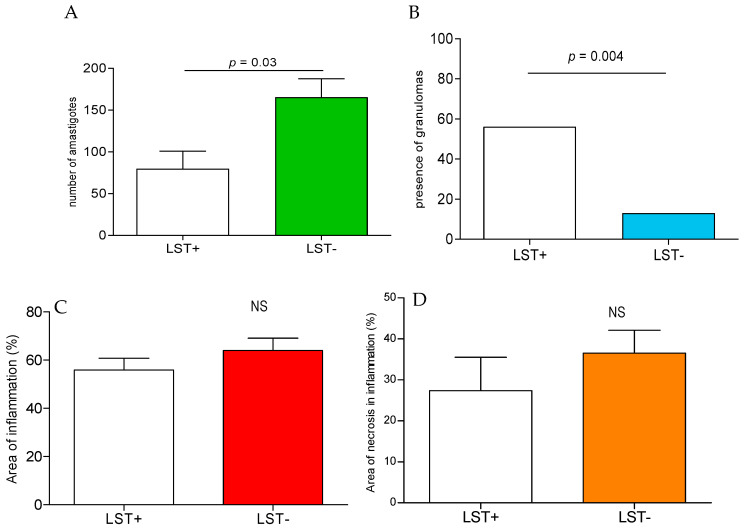
Histopathologic features of amastigotes. (**A**) Number of amastigotes. (**B**) Presence of granulomas. (**C**) Percentage of inflamed area. (**D**) Percentage of necrosis area in inflammation. These studies were performed in 26 LST+ and 26 LST- patients and unpaired T-test was used for statistical analysis. NS = not significant.

The histopathologic analysis with the quantification of amastigotes, number of granulomas, area of inflammation, and necrosis is shown in Figure 3. The cellular infiltrate in the areas of inflammation was concise with regard to macrophages, lymphocytes, and rare neutrophils, and was interspersed by foci areas of necrosis. The number of amastigotes detected by IHC in the LST- biopsies was higher (165.6 ± 91.2) when compared to LST+ (79.8 ± 55.6, *p* = 0.03 Figure 3A), and granulomas were more frequently seen in LST+ biopsies than LST- (56% vs. 15%, *p* = 0.004, respectively; see Figure 3B). Despite the impairment in the Th1 type of immune response, the areas of inflammation with necrosis were similar in LST+ and LST- (Figure 3C,D).

### 3.3. Immunological Profile

**Figure 4:**
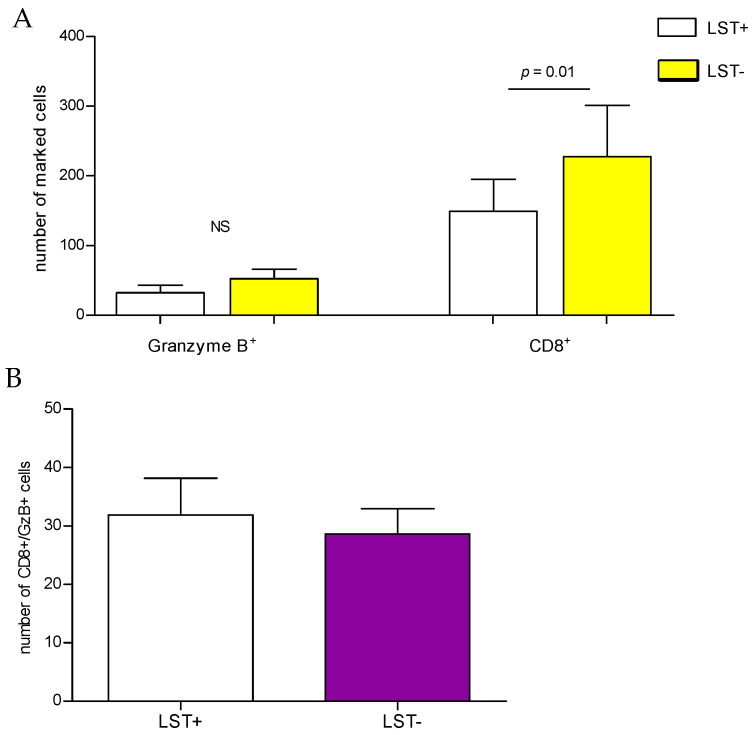
Granzyme B and CD8 immunostained cells. (**A**) Cells expressing GzmB and CD8 were identified by simple immunostaining. (**B**) Cells were identified simultaneously by double immunohistochemistry. This measure was obtained by analyzing 9 biopsies each of LST+ and LST-, respectively. Unpaired T-test was performed using GraphPad Prism 8 software. NS = not significant.

**Figure 5:**
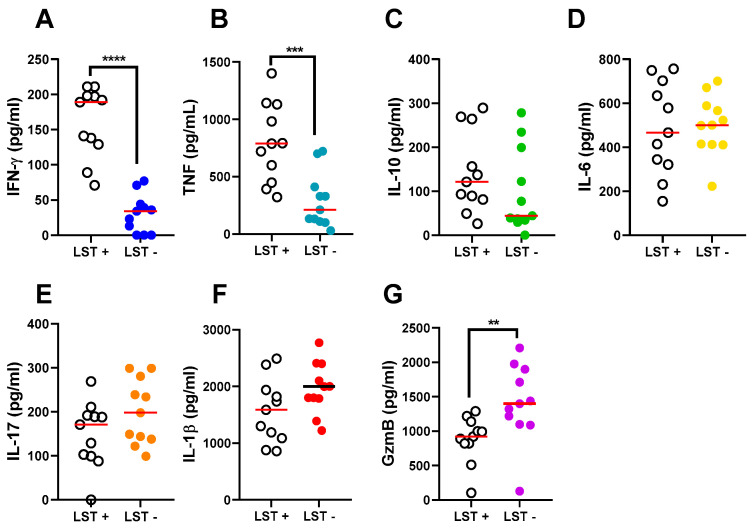
Production of cytokines in skin biopsies of LST-positive and LST-negative subjects with cutaneous leishmaniasis. Biopsies of L. braziliensis lesions from 11 LST+ and 11 LST- CL patients were cultured without stimulus for 72 h. Cytokine levels in lesions supernatants were measured by ELISA: (**A**) IFN-γ, (**B**) TNF, (**C**) IL-10, (**D**) IL-6, (**E**) IL-17, (**F**) IL-1β, and (**G**) Granzyme B. Central red lines represent median values. Statistical analysis was performed using Mann–Whitney test. ** *p* < 0.01, *** *p* < 0.0005 **** *p* < 0.0001 with GraphPad Prism 8 software. Abbreviations: CL, cutaneous leishmaniasis; ELISA, enzyme-linked immunosorbent assay; IFN-γ, interferon-γ; IL, interleukin; LST, *Leishmania* skin test; TNF, tumor necrosis factor; GzmB, Granzyme B.

The number of CD8+ T cells, the number of cells expressing GzmB, and the number of CD8+ GzmB+ T cells is shown in Figure 4. Granzyme B+ cell quantity was not different in the two groups despite the higher number in LST- than LST+ (Figure 4A). There were more CD8+ T cells in LST- than LST+ biopsies (227.6 ± 73.81 vs 149.0 ± 46.21, *p* = 0.01), and a similar number of CD8+ T cells expressing GzmB in the two groups (Figure 4B). To better characterize the immune response in LST- CL patients, the production of cytokines was determined in the supernatants of skin biopsies (Figure 5). As expected, we observed a lower production of IFN-γ (*p* < 0.0001) and TNF (*p* < 0.0005) in LST- compared with LST+ CL patients. There was no difference in IL-10 as well as in the IL-6, IL-17, and IL-1β levels among the two groups of patients. However, Granzyme B levels were higher in LST- than in LST+ CL patients (*p* < 0.01).

## 4. Discussion

A delayed hypersensitivity test for *Leishmania* antigens was widely used for the diagnosis of ATL before the advent of the PCR, but it continues to be used in highly endemic areas, where the large number of CL cases limits the use of histopathologic analysis and PCR. In the southeastern region of the state of Bahia, Brazil, an endemic area of CL, about 5% of patients may have a negative LST. Here, we evaluate a large number of patients with LST- to better characterize the clinical, histopathologic, and immunologic features of LST- CL patients, and to associate histopathologic and immunologic data with pathology.

The demographic characteristics were similar in the two groups. The predominance of young men with CL has been observed in previous studies [22,23]. The illness duration was higher in LST- than LST+ likely due to the delay among patients in recognizing the clinical manifestations as CL. In the initial phase of LST+ CL patients, a nodular lesion associated with a huge satellite lymph node is observed, and the ulcer only appears after 2–3 weeks [29]. However, LST- does not present satellite lymph nodes and the ulcers are large and may be atypical. The number of lesions did not differ in the two groups and none of the LST- patients developed disseminated leishmaniasis characterized by more than 10 papular, acneiform, and ulcerated lesions in at least two parts of the body [18]. Despite the impaired Th1 immune response, none of the LST- CL patients developed diffuse cutaneous leishmaniasis (DCL) characterized mainly by infiltrate nodules with macrophages full of amastigotes rather than ulcers [16]. The ulcer formation in CL is due to the host inflammatory response and as the ulcers in LST- were larger than in LST+, the inflammatory pathways related to the pathology are enhanced in LST-. The cure rate of CL is quite variable. In some endemic areas of *L. braziliensis*, transmission is very high [30], and here we confirmed that curing LST- had not only a high rate of failure in MA therapy, but there was also delayed healing time of the disease.

The cellular infiltrate in the lesion site of CL is characterized by an increase in the number of macrophages and lymphocytes, inflammation, granuloma formation, and a scarce number of parasites [31,32,33]. Macrophages and lymphocytes are the main cells in the granuloma, which is a cellular infiltration associated with a T-cell response that contributes to maintaining the parasites in the lesion site. Despite the decrease in the number of granulomas, parasites did not disseminate in LST-negative patients. However, as parasite killing is mediated by macrophages activated by IFN-γ, there were more parasites in LST-negative patients. Of note, the decrease in the Th1 immune response did not decrease the areas of inflammation and necrosis. This may be justified by the previous studies in mice and humans that have documented a pivotal role of CD8+ T cells and NK cells, rather than CD4+ T cells in tissue destruction and ulcer formation in *L. braziliensis* infection [8,20,21,34,35].

CD8+ T cells in CL have an impaired ability in parasite killing, but kill cells infected with *Leishmania* or expressing *Leishmania* antigens, which indicates they have a more cytolytic than protective profile [8,36]. As previous observations have indicated, CD8+ and GzmB-expressing cells participate in the pathology of CL lesions [17,18,25]; we have focused our studies on evaluating the frequency of these cells as well the frequency of CD8+ T cells expressing granzyme at the lesion site. While a similar number of cells expressing GzmB does not support the role of GzmB in the pathology of LST-, it cannot be ruled out that CD8+ T cells in LST- are more degranulating and release a large amount of GzmB, which consequently decreases the frequency of cells expressing GzmB. Moreover, in addition to CD8+ T cells, NK cells are important sources of GzmB. There is evidence that in addition to CD8+ T cells, NK cells participate in the cytolysis in CL [20,21,36].

The systemic immune response in CL patients evaluated by the cytokine profile in supernatants of PBMC has shown an increased production of proinflammatory and cytolytic cytokines, including IL-1β, IL-6, IL-17, and GzmB, respectively, but the production of these cytokines did not differ in LST- and LST+ CL patients [23]. However, there is evidence that the immune response at the lesion site differs from the one observed in peripheral blood, and the production of cytokines in situ is better associated with pathology than in PBMC [9]. The analysis of the cytokine production in supernatants of CL patient biopsies brought an important contribution to clarifying the immune response and the pathology in LST- CL patients. A poor production of IFN-γ in the skin explains the decreased ability of *Leishmania* killing and consequently the high parasite burden in LST- patients. While IFN-γ has a protective function in *Leishmania* infection, IL-10 facilitates *Leishmania* proliferation and dissemination [37,38]. However, there was no enhancement in the production of IL-10, indicating that the high parasite burden in these cases is likely due to poor IFN-γ production and decreased ability of *Leishmania* killing by macrophages. Recently, we documented a strong direct correlation between IL-1β, IL-17, and GzmB production in supernatants of CL biopsies and lesion size [9]. In both mice and humans, there is evidence of the pivotal role of GzmB, IL-17, IL-1β, and inflammasome formation in the pathology of *L. braziliensis* infection [39,40,41]. However, we did not document an enhanced production of either IL-1β or IL-17 at the lesion site of LST negative patients, suggesting that GzmB is the main cytokine contributing to pathology in this group of patients.

The study highlights that patient with a negative *Leishmania* skin test (LST-) exhibit larger and more atypical ulcers, a higher rate of treatment failure, and delayed healing, indicating a more severe clinical presentation and a need for alternative diagnostic and therapeutic approaches in endemic areas.

This study has several limitations mainly because we did not have data on cellular markers of degranulation of cytotoxic CD8+ T cells and of the frequency of NK cells at the lesion site. However, it brought relevant contributions to the understanding of the pathogenesis of the *L. braziliensis* infection and to the management of LST- CL patients. We showed that the pathology in LST- CL patients is mainly mediated by CD8+ T cells and GzmB. The high rate of therapeutic failure to MA in these patients indicates that they should be treated with more effective drugs such as Miltefosine or Amphotericin B.

## Figures and Tables

**Table 1:** Clinical features and outcomes of patients with cutaneous leishmaniasis and positive or negative *Leishmania* skin test status (LST).

Variables	LST+ (n = 67)	LST- (n = 67)	*p*-Value
Age (years), mean ± SD	28.3 ± 11.7	37.8 ± 14.4	0.13 *
Male no. (%)	40 (59.7)	38 (56.7)	0.86 ^#^
Illness duration (days), median ± SD	37 ± 17.8	57 ± 35.8	0.0001 *
Number of lesions	1 (1–8)	1 (1–8)	0.08 *
Area of lesion (mm^2^), mean ± SD	226 ± 245.4	1090 ± 3073.9	0.02 *
^#^ Cure rate on day 90, no. (%)	44 (65.7)	22 (43.3)	0.004 *
Healing time (days,) mean ± SD	78.7 ± 53.9	126.32 ± 90.29	0.0006 *

* Unpaired T-test; ^#^ Fisher’s exact test; no. = number.

## Data Availability

The data presented in this study are available upon request to the corresponding authors due to patient data protection and ethical restrictions.

## Supplementary Materials

The following supporting information can be downloaded at https://www.mdpi.com/article/10.3390/pathogens13111018/s1, Data S1: Flow chart for the handling of patients; Data S2: Table of Cutaneous Leishmaniasis treatment protocols and cure evaluation.
