# Supplementary material for: The Leishmania Skin Test Predicts Clinic-Immunologic and Therapeutic Outcomes in Cutaneous Leishmaniasis
Source: Pathogens. 2024 Nov 19;13(11):1018. doi: 10.3390/pathogens13111018 (PMC11597541; doi:10.3390/pathogens13111018)
Supplement: Supplementary file 1 [file pathogens-13-01018-s001.zip › pathogens-3280860-supplementary.pdf]

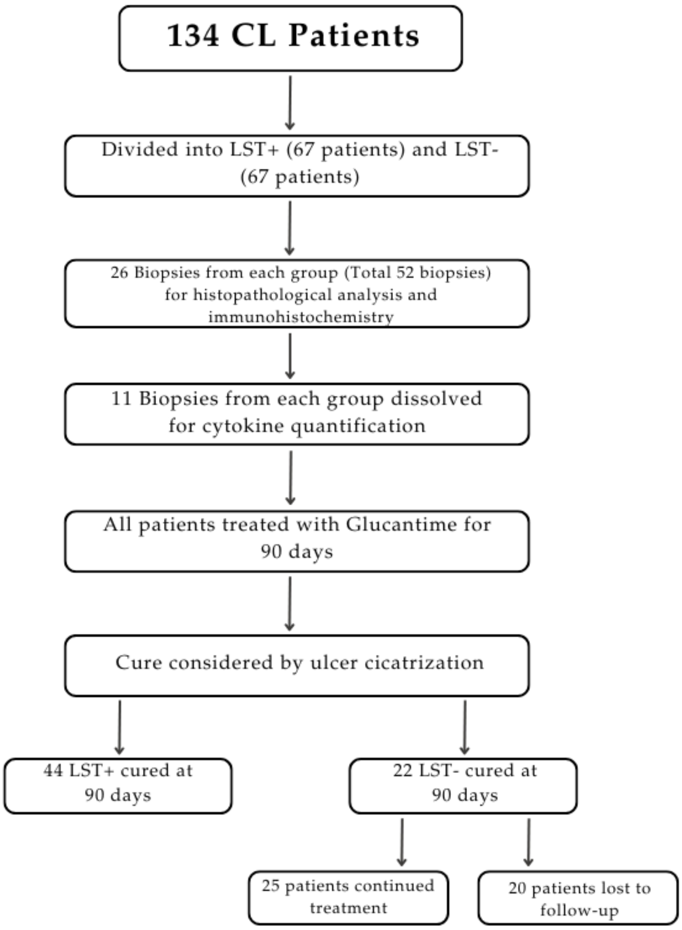

Supplementary data 1. Flow chart for the handling of patients.

Treatment Protocol	Details
Medication	Glucantime (Sanofi Aventis)
Dosage	20mg/kg/day
Duration (initial course)	20 days
Evaluation Frequency	Every 30 days
Cure assessment	90 days after therapy initiation
Cure definition	Complete reepithelization of lesions, without raised borders on day 90
If not cured (after initial course)	Additional course of Glucantime for 30 days
If still not cured (after second course)	Continue Glucantime for an additional 30 days
If not cured (after third course)	Miltefosine 2,5mg/kg for 20 days
If not cured	Treatment with Amphotericin B 3-5mg/kg/day (Total dose 30mg/kg)

Supplementary data 2. Table of Cutaneous Leishmaniasis treatment protocols and cure evaluation.
